# Voyages in Vacation. II

**Published:** 1888-10-06

**Authors:** 


### Voyages in Vacation.—II.

By One in Search of Restful Restoratives.

(*Continued from page* 419, *No.* 105, *Vol. IV.*)

### PLEASURE CRUISES AVAILABLE.

We have already explained that there are already some yachts which are specially fitted to carry passengers for a circular tour, according to the season of the year—north, south, and west. It is not surprising to find an old P. and O. steamer regarded by many as too old for complete comfort. Rats and cockroaches are not pleasant travelling companions, but in old vessels there must necessarily be some, and often many, such passengers, whilst the long wear and exposure to which the planking has been subjected renders it difficult, if not impossible, to keep the cabins watertight. Yet wet and damp are two worse evils than cockroaches and rats, so that any pleasure yacht which is not free from all four drawbacks can hardly hope to be entirely popular and successful.

The *Victoria* is a new boat, has not a rat on board, and is as watertight as a life-boat. She is, besides, a good sea boat, and has probably not an equal in these respects. Those who have been fortunate enough to travel in the *Victoria* will also know how great a comfort it is to have a captain like Commander R. D. Lunham, F.R.G.S., who is every inch a seaman, and who knows his work and does it in a way to leave nothing to be desired. These pleasure yachts, as at present conducted, have, however, one very serious drawback, which must render them doubtful financial ventures so long as it is allowed to continue. Life on an ordinary ocean steamer is probably the most cosmopolitan and free that can well be imagined. The ship has one head, and only one—the commander—who looks after and is responsible for everything connected with it. On the pleasure yachts this is not always the case. Some are owned by private individuals, who have selected the most comfortable portions of the vessels for their private accommodation, and who nearly always occupy these cabins. If they have any special plans to carry out, it is not unnatural, perhaps, that they should make their own convenience the first consideration, and so interfere with the working of the ship, the regulations laid down by the commander, and the convenience and comfort of the passengers. For example, to appropriate the steam launch for the owner's use when in port, or to alter the course of a cruise for private reasons in breach of the arrangements and posted regulations, are very improper acts on the part of the owners, and ought not to be allowed for one moment. Yet these and similar acts may not only be committed by the owners, but the passengers may often be seriously annoyed by the owner's conduct in other ways. These yachts can never be entirely satisfactory or successful until the captain is left in supreme and unfettered command. If the owner accompanies his yacht from time to time he should travel as a passenger, and as a passenger only; and it would be a good day for everybody were a pleasure yacht to be started where the owner did not interfere and where he was always left ashore. No one who has sailed in one of the existing pleasure yachts can doubt the force or justice of this contention, and those who have yet to try this mode of travel would be well advised to make inquiries on this point before taking passage in any vessel.

The steam yacht excursions have been planned with considerable judgment. The pleasure seeker has a great variety of choice offered for his enjoyment. If he desires to take his holiday in February, he can visit the Mediterranean and Black Seas—a cruise which will take about seventy-five days. If the Black Sea portion be omitted, about six weeks may be pleasantly occupied by a cruise in the Mediterranean, in the course of which Lisbon, Malaga for Granada and the Alhambra, Villefranche for Nice and Monte Carlo, Genoa, Naples, Palermo, Piræus, Malta, Tunis, Algiers, Gibraltar, and Tangiers will be visited, ample time being allowed to see each of these places comfortably. The cost of the longer cruise would be £150 to £175 for the seventy-five days, and of the shorter one (six weeks) £80 to £100. This cost includes wines (claret and sherry), the use of the steam launch when in port, and all other charges. If April be the more convenient month, then a visit may be paid to the Azores, Canary Islands, Madeira, Gibraltar, and Lisbon, at a cost of about £60 for the thirty days. In the months of June, July, and August the Norwegian fiords or the North Cape and the Land of the Midnight Sun offer the tourist most tempting prospects for pleasure and health. Four such excursions are usually made by the *Victoria* during these months, lasting from sixteen to twenty-five days, the expense varying from £32 to £50 according to the time occupied and number of places visited. In September, a voyage may be profitably made to the Baltic, including Copenhagen, St. Petersburg, Moscow, Stockholm, Gottenburg, Gotha Canal, and Christiania, lasting about a month, and costing from £60 to £75 inclusive, according to whether a single or double cabin be taken. The latter months of the year may be utilized to visit the Mediterranean or India. Those who prefer a longer and wider experience than any of the foregoing offer will be glad to join the *Victoria* on February 9th, 1889, and to make an excursion to the West Indies occupying seventy-five days. This is declared to be the most enjoyable of all excursions by steamer, as it includes a visit to Madeira, Teneriffe, Trinidad, Grenada, Barbadoes, Port Royal and St. Pierre (Martinique), Dominica, Guadaloupe, St. Thomas, San Domingo, Jamaica, Santiago de Cuba, Havana, Nassau (Bahamas), Bermuda, and the Azores, so that the very best West Indian and island scenery will be visited. The inclusive cost will probably not exceed £150 for a single berth, or £175 for a single cabin. A cruise round the world has been often talked of, but not yet carried to a successful issue, and the probable reason is not far to seek. We shall refer to it, however, in another article, because it involves many questions of policy and arrangement which are of the first importance to travellers in pleasure yachts.

The outline we have given of the various cruises now placed within the easy reach of everybody who has a laudable ambition to widen his area of vision, to enlarge his sympathies, and to ripen his judgment by seeing the world, will no doubt suffice to prove how great an impetus to travel these pleasure yachts have afforded. There is no method of transit so pleasant or comfortable for the traveller as that afforded by a well-found ocean-going steamer of the first class. Our intercourse with America has brought the ocean steamer to a pitch of perfection which leaves little, if anything, to be desired. The popular prejudice against sea travel must gradually disappear, as experience proves to more and more people that to the average traveller with normal health and fair powers of self control, a journey by steamer is neither conducive to sea-sickness nor anything but the most comfortable of all travelling experiences. We shall hope to show later how sea-sickness can be rendered so slight as to be unimportant, if it cannot be obviated altogether by the majority who are wise enough to follow faithfully a few simple rules of life. We have now crossed many seas in all sorts of weather, and are therefore entitled to claim that our contention is not based upon any mere landsman's theories, but upon the practical experience of one who has sailed the seas and done business in the great waters.

(*To be continued.*)

